# Sleep quality and common mental disorder in the hospital Nursing team*

**DOI:** 10.1590/1518-8345.4280.3444

**Published:** 2021-08-30

**Authors:** Jolana Cristina Cavalheiri, Claudicéia Risso Pascotto, Nelsi Salete Tonini, Ana Paula Vieira, Lirane Elize Defante Ferreto, Franciele Ani Caovilla Follador

**Affiliations:** 1 Universidade Paranaense, Francisco Beltrão, PR, Brazil.; 2 Universidade Estadual do Oeste do Paraná, Francisco Beltrão, PR, Brazil.; 3 Universidade Estadual do Oeste do Paraná, Cascavel, PR, Brazil.

**Keywords:** Nurse Practitioners, Occupational Health Nursing, Occupational Health, Sleep, Mental Disorders, Hospitals, Profissionais de Enfermagem, Enfermagem do Trabalho, Saúde do Trabalhador, Sono, Transtornos Mentais, Hospitais, Enfermeras Practicantes, Enfermería del Trabajo, Salud Laboral, Sueño, Trastornos Mentales, Hospitales

## Abstract

**Objective:**

to determine the prevalence of sleep quality and common mental disorder in Nursing professionals and factors associated with sleep change.

**Method:**

a cross-sectional, analytical and quantitative study developed with 196 Nursing professionals of a public hospital and a mixed one. Data was collected by means of an instrument of sociodemographic characterization, by the Self-Report Questionnaire 20 and Pittsburgh Sleep Quality Index and were analyzed by descriptive and inferential statistics to identify possible factors associated with sleep changes.

**Results:**

sleep changes were identified among the Nursing professionals with a frequency of 76.5% (70.4-82.1). Sleep quality was classified as poor in 41.8% (41.8-55.6) and sleep disorder in 27.6%. (21.4-34.2). The prevalence of common mental disorder was identified in 36.7% (30.1-43.9). The main factor for poor sleep quality was the presence of common mental disorder (Odds Ratio: 5.15; p<0.001).

**Conclusion:**

sleep changes were prevalent and the characteristics of the work environment and the presence of mental disorder showed relevance in the changes.

## Abstract (pt)

**Objetivo:**

determinar a prevalência da qualidade do sono e do transtorno mental comum em profissionais de enfermagem e os fatores associados à alteração do sono.

**Método:**

estudo transversal, analítico e quantitativo desenvolvido com 196 profissionais de enfermagem de um hospital público e um misto. Os dados foram coletados por meio de instrumento de caracterização sociodemográfica, do *Self-Report Questionnaire* 20 e do Índice de Qualidade de Sono de Pittsburgh e foram analisados por estatística descritiva e inferencial para identificar possíveis fatores associados com a alteração no sono.

**Resultados:**

a alteração do sono foi identificada entre os profissionais de enfermagem numa frequência de 76,5% (70,4-82,1). A qualidade do sono foi classificada como ruim em 41,8% (41,8-55,6) e o distúrbio do sono em 27,6% (21,4-34,2). Identificou-se uma prevalência de transtorno mental comum em 36,7% (30,1-43,9). O principal fator para a má qualidade do sono foi a presença de transtorno mental comum (*Odds ratio*: 5,15 p<0,001).

**Conclusão:**

as alterações de sono foram prevalentes e as características do ambiente de trabalho e a presença de transtorno mental comum apresentaram relevância com as alterações.

## Abstract (es)

**Objetivo:**

determinar la prevalencia de la calidad del sueño y del trastorno mental común en los profesionales de enfermería y factores asociados a los trastornos del sueño.

**Método:**

se trata de un estudio transversal, analítico y cuantitativo desarrollado con 196 profesionales de enfermería de un hospital público y otro mixto. Los datos se recolectaron mediante un instrumento de caracterización sociodemográfica, utilizando el *Self-Report Questionnaire* 20 y el Índice de Calidad de Sueño de Pittsburgh y se analizaron utilizando estadísticas descriptivas e inferenciales para identificar posibles factores asociados con trastornos del sueño.

**Resultados:**

se identificaron trastornos del sueño entre los profesionales de enfermería con una frecuencia del 76,5% (70,4-82,1). La calidad del sueño se clasificó como mala en el 41,8% (41,8-55,6) y trastorno del sueño en el 27,6% (21,434,2). Se identificó una prevalencia de trastorno mental común en el 36,7% (30,1-43,9). El factor principal de la mala calidad del sueño fue la presencia de trastorno mental común (*Odds ratio*: 5,15 p<0,001).

**Conclusión:**

los trastornos del sueño fueron prevalentes y las características del entorno laboral y la presencia de trastorno mental común fueron relevantes para los trastornos del sueño.

## Introduction

Work is an important factor for human life and society development as it allows purchasing goods, as well as it influences the physical and mental health of the individual^(1-2)^. However, with globalization, the incorporation of technologies, production demand, qualification and the new ways of management, important changes were observed in the work dynamics of several sectors of society^(3)^.

These transformations impacted workers’ health, as many national and international studies have been demonstrating that the work activity has an effect on professional illnesses^(4-7)^. Situations such as work fragmentation, excessive workload, complexity of the service, accumulation of functions to reach the goals, as well as low wages and the terrible working conditions are organizational features that can negatively intervene in the professional’s health and quality of life^(8)^. In the hospital environment, these situations add up to the contact with at risk patients and to the need to make important decisions^(9)^, which turn this place into one of the most complex health services.

Thus, the largest workforce in the hospital environment is the Nursing team, which remains with the patient 24 hours a day and offers continuous care. In this profession, the work process is developed collectively and is fragmented into three categories: the Nursing assistant and technician, who perform hygiene and comfort tasks, and less complex procedures and the nurse, who acts as team supervisor and service manager^(1)^.

These particularities of each category, along with the organization of the hospital’s work process, are related to the development of work-associated health changes^(3)^. Among them, the complaints on poor sleep quality are common, especially in the individuals that work in the night shift^(4)^.

It is known that sleep deprivation favors daytime sleepiness, diminishes the alert state, increases the chance of injuries and work accidents, contributes to cognitive overload, reduces performance in the activities and analytical reasoning, leads to cognitive failure and is associated with two times more errors by the Nursing team^(9–10)^, being considered one of the main threats to patient safety in hospital units^(4)^.

Sleep impairment and wearing work show relevance in the mental and physical health of the professionals, with worsening in quality of life, irritability, chronic fatigue, anxiety, depression, tiredness and development of mental disorders^(5,11-12)^. Among them, there is minor or common mental disorder (CMD; *transtorno mental comum-TMC*-in Portuguese language), a less severe group of psychiatric disorders, which involves loss of concentration, forgetfulness, somatic complaints, and fatigue^(13)^, being associated with the characteristics of the work environment^(6,9,14)^.

It is to be pointed out that, in a study developed in Iran with nurses in hospital units, the prevalence of professionals with poor sleep was obtained^(10)^, as well as a research study conducted in a general hospital in the state of Bahia (Brazil), in which the professionals presented sleepiness and insomnia as common complaints^(6)^. In addition, in a study developed in the state of Paraná (Brazil) it was found that the professionals of the night shift, who are dissatisfied with sleep, presented increased chances of emotional exhaustion^(7)^.

In face of this issue which involves the Nursing team, it is important to know the changes in sleep and mood experienced by the professionals and their relationship with the work environment, in order to propose changes in the hospital that minimize the negative effects of work on health and improve the quality of life of the professionals. Thus, the guiding questions of the study were as follows: What is the prevalence of mental and sleep changes in Nursing professionals in the hospital environment? And what factors are associated with poor sleep quality?

From this assumption, this research aimed to determine the prevalence of sleep quality and common mental disorder in Nursing professionals and factors associated with sleep change.

## Method

This is a cross-sectional, analytical, and quantitative study developed with assistance Nursing professionals from a public hospital, and from another mixed one, in Francisco Beltrão, Paraná (PR), Brazil.

The Human Development Index for the aforementioned municipality is 0.774, and this city is a health care hub for the population of Southeast of the State of Paraná, hosting the 8^th^ Health Region. The health sector in the municipality manages the operation of care through a network composed of basic, specialized and tertiary units. The basic network covers 75.07% of the population. As for the tertiary units, they correspond to four hospitals, one managed by the State, one private and two with mixed management (public/private), which have 476 Nursing professionals altogether.

Two tertiary units were selected for the study. The first, a mixed hospital unit, with private service, under agreements with the Unified Health System (UHS; SUS is the name given in Brazil to the public health system), with 72 beds. Care is directed to the following specialties: general surgery, medical clinic, obstetrics, pediatrics, and it also has an Adult Intense Care Unit (10 beds). The second is a State-managed, tertiary unit, which serves only the UHS. It can serve medium- and high-complexity cases, with 108 beds, offering services in the following specialties: trauma orthopedics, surgical and medical clinic, obstetrics, pediatrics, and psychiatry. It features an Adult Intensive Unit Care (10 beds), a Neonatal Intensive Care Unit (10 beds), and a Conventional Neonatal Intermediate Care Unit (5 beds).

**Figure 1:**
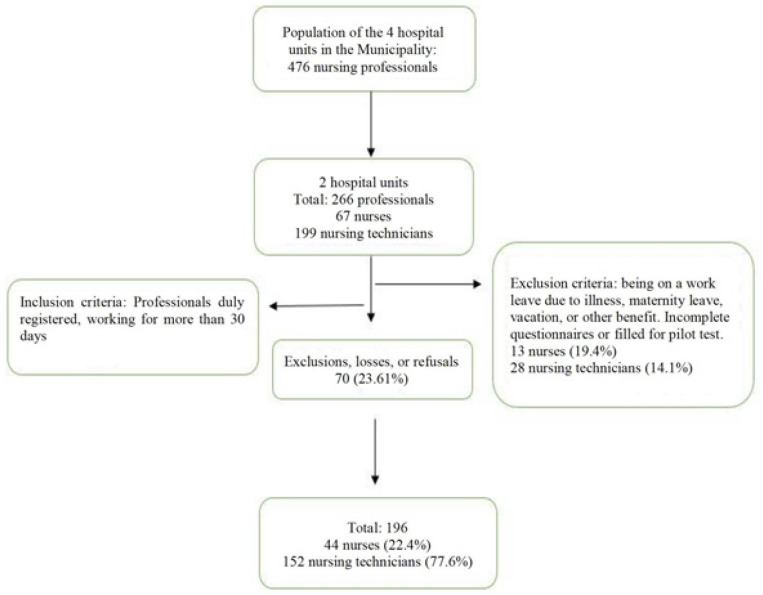
Flowchart of the sample selection. Francisco Beltrão, PR, Brazil, 2018

These places were chosen for the study due to their very similar characteristics in serving the population, but with differences in hospital management. All the Nursing professionals who work in both hospital units were invited to participate in the research, totaling 266 workers. Of this total, 196 Nursing professionals participated in the study: 44 nurses (22.4%) and 152 nursing technicians (77.6%) (Figure 1). The participants performed tasks in both institutions and were included in the study only once.

The data were collected from February to August 2018 by filling in self-response questionnaires, in which the professionals, first of all, received guidelines for their completion during the shift break periods or at the beginning and end of each shift, with any possible doubt being clarified. Aiming at greater adherence by the professionals and to avoid interruptions of their assistance activities, the questionnaires were handed in with a 10day return deadline.

With an organizational method of data collection, it was chosen to divide the instruments into blocks, the first being a questionnaire composed of sixteen closed questions that contemplated the sociodemographic and occupation data elaborated by the researchers, according to the national and international literature. The studied variables were age, gender, schooling, marital status, self-referred skin color, family income, professional category, work period, academic training, time working in the profession and at the institution, if they had more than one job, and weekly working hours. A pilot test was carried out to clarify possible doubts, making the instrument available to 5 Nursing professionals of each institution, from various units. There was no need to adapt the survey questionnaire and the pilot test respondents were excluded from the final sample.

As for the second block, it aimed to assess mental health, in which the SRQ-20 (Self-Report Questionnaire) Mental Disorders Assessment Questionnaire was used, validated in Brazil in 1986, which is composed of 20 questions divided into characteristic symptoms of depressive-anxious and somatic mood, decreased vital energy and depressive thoughts. The alternatives were dichotomized into affirmative and negative. This has the objective of tracking non-psychotic disorders^(15)^. The instrument score varies from 0 to 20 points, with values ≥ 7 indicating the possibility of common mental disorder (CMD).

Finally, the third assessment block consisted of the verification of sleep quality, in which the Pittsburgh Sleep Quality Index (PSQI) was used, validated in Brazil in 2008. This instrument assesses the perception of sleep in the last 30 days and is composed of 19 self-applied questions. These questions are grouped into seven components: subjective sleep quality, sleep latency, sleep duration, usual sleep effectiveness, sleep disorders, use of medications to sleep and daytime dysfunction. The global score varies from 0 to 21, with values under 4 indicating good quality; from 5 to 9 points, poor quality and over 10 points, presence of sleep disorders^(16)^.

The data were inserted into a Microsoft Excel for Windows/7 (Microsoft Office 2007) spreadsheet and later explored in the IBM SPSS Statistics statistical package, version 21 and in the Minitab Statistical Software, version 16. The absolute (n) and relative (%) frequencies were used to describe the characteristics of the sample.

Initially, the Pittsburgh Sleep Quality Index was categorized into absence (a score below 4, considered good sleep quality) and presence (higher than 5, considered as poor sleep quality and sleep disorder) of sleep changes. Immediately after that, for a comparison between presence or absence of sleep changes and the categorical variables, the chi-square test with continuity correlation was performed. The variables that presented p<0.25 in this analysis were inserted in the binary logistic regression model to identify possible factors associated with sleep changes, with values that presented p<0.05 being considered statically significant. The model correctly classified 65.8% of the cases and explained 10.1% of the variance (Cox and Snell R^2^). The assumption of normal distribution was tested by the Kolmogorov-Smirnov test*.* Considering that the distribution did not meet the assumptions of the parametric statistics, the non-parametric Kruskal-Wallis test was used to verify the relationship between the independent variables and the scores of the sleep instruments, only the variables with statistical significance (p<0.05) being presented (Table 3).

Thus, this study followed the ethical recommendations in research with human beings and, initially, authorization was requested from the hospital institutions for the research to be carried out. Subsequently, it was submitted to the Ethics and Research Committee of the State University of Western Paraná and approved on December 4^th^, 2017, under opinion No. 2.415.008.

## Results

With this research it was possible to analyze that, among the 196 Nursing professionals, there was a predominance of nursing technicians (78.7%), females (88%), aged over 36 years old (58.7%) and receiving more than 3 minimum wages (71.3%).

Sleep changes were identified among the Nursing professionals with a frequency of 76.5% (n=150) (95% CI: 70.4-82.1). Sleep quality was classified as poor in 41.8% (n=96) (95% CI: 41.8-55.6), followed by sleep disorder in 27.6% (n=54) (95% CI: 21.434.2). A prevalence of common mental disorder was identified in 36.7% (n=72) (95% CI: 30.1-43.9). The sociodemographic, occupational and CMD characteristics are in the table presented in continuity (Table 1).

**Table 1:** Sociodemographic and occupational characterization, common mental disorder and sleep changes among Nursing professionals (n=196). Francisco Beltrão, PR, Brazil, 2018

Variables	Sleep Changes				p-value
	Presence		Absence		
	(N=150)	(%)	(N=46)	(%)	
Professional category					0.635
Nurses	32	21.3	12	26.1	
Nursing Technicians	118	78.7	34	73.9	
Gender					1.000
Female	132	88	41	89.1	
Male	18	12	05	20.9	
Age group (years old)					0.091
18 to 35	62	41.3	12	26.1	
Over 36	88	58.7	34	73.9	
Skin color					0.545
White	119	79.3	39	84.8	
Brown/black	31	20.7	07	15.2	
Schooling					0.702
Technical education	91	60.7	30	65.2	
Higher education or above	59	39.3	16	34.8	
Marital status					0.083
Single	59	39.3	11	23.9	
Married/Stable union	91	60.7	35	76.1	
Family income (minimum wages^*^)					0.878
Less than 3	43	28.7	12	26.1	
More than 3	107	71.3	34	73.9	
Time in the profession (years)					0.480
Up to 10	86	57.3	23	50	
Over 10	64	42.7	23	50	
Hospital Unit					1.000
Public	100	66.7	31	67.4	
Private/Public	50	33.3	15	32.6	
Time working in the institution (years)					0.783
6 or less	50	33.3	17	37	
Over 6	100	66.7	29	63	
Number of jobs					0.562
Only 1	116	77.3	33	71.7	
2 or more	34	22.7	13	28.3	
Number of weekly working hours					1.000
Up to 40	87	58	25	54.3	
41 or more	63	42	21	45.7	
Employment contract					1.000
Statutory	101	67.3	31	67.4	
Service provision	49	32.7	15	32.6	
Working shift					0.357
Day	95	63.3	25	54.3	
Night	55	36.7	21	45.7	
Have worked in the night shift					0.430
Yes	117	78.0	39	84.8	
Never	33	22.0	07	15.2	
Common Mental Disorder					<0.001
No	84	56	40	87	
Yes	66	44	06	13	

* Brazilian minimum wage in 2018: R$ 954.00 (nine hundred and fifty-four reais)

Regarding the variables in Table 1, it is observed that age group, marital status and common mental disorder presented p<0.25 and were inserted in the logistic regression model to identify factors associated with sleep changes (Table 2). However, only one of the three variables presented statistical significance: common mental disorder. Thus, the Nursing professionals who were classified with CMD presented a 5 times higher probability of sleep changes (p-value<0.001).

**Table 2:** Factors associated with the presence of sleep changes in Nursing professionals in Francisco Beltrão, PR, 2018

Variable	grOR^*^(95% CI^†^)	p-value	**adOR^‡^(95% CI)**	p-value
Age group (years old)				
18 to 35	1			
Over 35	1.99 (0.95−4.15)	0.091	-------	
Marital Status No partner Has a partner	1 2.06 (0.97−4.37)	0.083	-----	
Common mental disorder Absence Presence	1 5.23 (2.09−13.1)	<0.001	1 5.15 (2.04−12.90)	<0.001

* grOR = Odds Ratio;

† CI = 95% Confidence Interval;

‡ adOR = Odds Ratio (adjusted measures)

The mean time to fall asleep was 31.9 minutes, with a mean of 6.5 sleep hours. Regarding the perception of sleep quality, 32.1% referred having good sleep; 31.1%, poor; 18.9%, very good and 17.9%, very poor. The consumption of sleeping medications was mentioned by 33.2% of the participants.

When the sociodemographic and occupational characteristics were assessed with the domains of the scores presented statistical significance with some variable, in which the public hospital obtained higher mean scores in subjective sleep quality, sleep disorder, use of sleep medications and daytime dysfunction. It was also verified that a working time of more than 6 years at the institution presented statistical significance in subjective sleep quality, sleep latency, sleep disorders and use of sleep medications, according to Table 3.

**Table 3:** Mean scores of the sociodemographic and occupational variables with the domains of the Pittsburgh Sleep Quality Index of Nursing professionals (n=196). Francisco Beltrão, PR, Brazil, 2018

		Subjective quality		Sleep latency		Sleep duration		Usual effectiveness		Sleep Disorders		Medication use		Daytime dysfunction	
Variable	Description	Mean Score	p-value	Mean Score	p-value	Mean Score	p-value	Mean Score	p-value	Mean Score	p-value	Mean Score	p-value	Mean Score	p-value
Hospital	Public	103.9	0.030	103.3	0.080	100.1	0.550	96.3	0.370	105.4	<0.001	103.5	0.030	104.9	0.010
	Private	87.5		88.8		95.3		102.9		84.6		88.5		85.5	
Gender	Female	98.9	0.790	98.9	0.750	97.4	0.450	98.8	0.810	99.9	0.290	100.9	0.040	98.9	0.800
	Male	95.8		95.2		106.5		96.2		88.1		80.6		95.8	
Skin color	White	96.9	0.370	97.4	0.540	98.6	0.950	102.7	0.010	97.3	0.510	96.7	0.270	99.6	0.540
	Black	105.2		103.3		98.1		81.2		103.4		106.1		93.7	
Number of jobs	Only 1	100.5	0.340	104.3	<0.001	100.6	0.320	94.6	0.040	99.7	0.540	102.3	0.040	96.8	0.430
	2 or more	92.2		80.1		91.7		110.9		94.6		86.3		103.9	
Working Hours	Up to 40	98.7	0.890	103.4	0.140	98	0.880	92.2	0.030	98.9	0.890	103	0.1270	94.8	0.270
	More than 40	97.9		91.9		99.2		107		98		92.5		103.4	
Work Shift	Day	97.8	0.790	102.5	0.200	108	<0.001	100.5	0.480	97.3	0.710	101.4	0.280	93.4	0.090
	Night	99.7		92.3		83.5		95.4		100.4		93.9		106.6	
Time working (years)	Up to 6	82.7	<0.001	85.6	0.010	99.3	0.870	104.8	0.190	81.7	<0.001	84	<0.001	90.5	0.130
	Over 6	106.7		105.2		98.1		95.2		107.2		106		102.7	

## Discussion

A number of studies have demonstrated that the work dynamics of the Nursing team generates high absenteeism rates, with implications on the physical and mental health of these professionals, who are responsible for the provision of comprehensive care and management aspects in the hospital units. In this research, it was sought to investigate two aspects that interfere with the occupational quality of the Nursing professionals and that, in general, contribute to their distancing from the service. Such aspects are the presence of minor mental disorders represented by CMD and sleep quality^(17)^.

In this study, it was verified that the characteristics of the Nursing professionals are similar to those of other research studies in Brazilian hospital units^(1,7,9,18)^. These data were also observed in a study developed in a university hospital in the state of Minas Gerais, where 87.9% of the professionals were women, with a mean age of 40.2 years old^(19)^. Another study conducted with Nursing technicians in Montes Claros, Minas Gerais, indicated that 58.6% were female, with a mean age of 38.5 years old^(20)^. A study developed with 540 nurses in 6 hospital institutions in Iran found that 66.3% were women with a mean age of 32 years old^(11)^, data similar to those in this research.

In relation to income, most of the professionals reported earning from three to five minimum wages, having only one job and working up to 40 hours during the week, which is similar to a research study carried out in the North of Paraná (Brazil), in which professionals had only one job (56.7%) and earned from one to two minimum wages^(7)^. Likewise, another study developed in China, with nurses working in different hospital units, presented 4,185 female professionals, with only one job and who worked up to 45 weekly hours^(21)^.

It is to be noted that 66.8% of the professionals had employment contracts after passing a public tender, similarly to the National Research of the Nursing Profile, which indicated 65.3% of the Brazilian professionals working in this service^(18)^. However, the type of contract tends to vary according to the economic scenario of each region, as a study conducted in the Northeast of Brazil found that service provision was the main hiring method^(1)^.

It was also observed that 76.5% of the professionals presented some sleep change, with 41.8% being classified as poor sleep, followed by presence of sleep disorders. Such data were similar to the study in the state of Rio Grande do Norte (Brazil), where 60.94% of the day shift nurses and 85% of the night shift professionals presented poor sleep quality^(22)^. A research study carried out in an intensive unit in Paraíba (Brazil), obtained 88.24% of the Nursing team members with sleep changes (classified as poor)^(23)^.

Similarly, a study in hospital units in Iran obtained 77.4% (418) of the nurses considered as with poor sleep^(11)^. Another study, developed in China with 4,730 nurses working in emergency units, found 65.8% of the professionals with altered sleep^(21)^. This finding is similar to the study conducted in India, which obtained 83.2% of the nurses as with poor sleep^(10)^. However, a research study developed in a hospital service in Colombia pointed out only 24.9% of the professionals with sleep changes, despite sleeping less than 7 hours a day^(24)^.

As for a study carried in hospital from the city of Larestan, in the South of Iran, to assess the prevalence and consequence of sleep changes in nurses, it obtained a mean score of 6.52 ± 4.23 in the sleep quality index. And, according to this index, 56% of the nurses were classified as with poor sleep; therefore, 78.5% were sleepy, 16.5% very sleepy and 5% severely sleepy during labor activities. In addition, the sleep disorders in the professionals who worked in the surgical section were higher than those in the nurses from other hospital units (p<0.05)^(25)^.

Another study conducted with nurses in the South of Italy has found that women presented worse sleep quality when compared to men, in addition to having less social support at work, which was negatively associated with sleep disorders^(26)^, while a study carried to assess the prevalence and other factors associated with sleep disorder in nurses (n=422) who work in federal government hospitals in Ethiopia, in Adis Abeba, obtained 41.8% of the participants with sleep changes, especially insomnia, in which work in threeshift rotations, (adjusted OR = 3.1, 95% CI: 1.68 to 5.83) was significantly associated with sleep disorder^(27)^.

These differences in the sleep classification using the same research instruments are associated with lifestyle, workload, the physiological stress of each individual^(28)^, gender, age, presence of children, the personality of each professional and the economic conditions of the country^(1)^. In addition to that, the exposure to environmental factors and to work dynamics with high workload and stress is associated with the development of sleep changes^(3,5,11,19)^.

Among the work-associated psychic disorders, CMD develops in different populations, especially in the professions that presented great requirements in the tasks fulfilled^(24)^. This study identified a CMD global prevalence of 36.7%, similar to a study conducted in a public hospital in the state of Bahia (Brazil), which found 35% of the Nursing professionals with CMD^(6)^, as well as in a research study carried out in a psychiatric hospital with the Nursing team, which obtained 25.7% as suspected cases^(29)^.

In a study conducted with nursing technicians in a university hospital in the state of Minas Gerais (Brazil), a CMD prevalence of 46.9% was observed^(20)^. High rates of mental disorders are worrisome in the hospital environment, as they have direct implications on the quality of the care provided to the patients under the Nursing team responsibility^(29)^.

As for the factors associated with sleep changes, the Nursing professionals who were classified with CMD had five times more probabilities of having poor sleep. This fact corroborates a meta-analysis carried out on sleep dissatisfaction and the development of psychic disorders, which found a strong relationship between insomnia and mental changes, including depression, anxiety and suicidal ideation^(30)^.

In a study conducted only with women, the association between CMD and shorter sleep duration was also obtained, pointing out that these changes are the first symptoms detected in psychic disorders. It is highlighted that the women who reported sleeping six hours or less presented 2.66 more chances of CMD when compared to those who slept more than seven hours^(31)^. A research study conducted with Chinese nurses also obtained an association between the presence of anxiety signs (OR = 8.07, IC 95%: 2.92-22.33) and poor sleep quality, long latency and insomnia^(32)^.

Likewise, a study also conducted in China with 1,500 nurses in six hospitals from the Shandong province associated the existence of depression symptoms with the presence of sleep disorders and, consequently, with lesser control of the work environment. Nurses are susceptible to the development of work-related mental disorders, thus impairing the quality of the care provided^(21)^.

It is also asserted that exposure to stress and poor sleep quality also relate to cognitive failures, such as memory errors, perception, planning, performance of tasks that occur on a daily basis and service provision^(33-34)^. These data are relevant because, in this study, most of the participants were women and reported sleeping 6.5 hours, which can contribute to accidents and risk behaviors. In the context of Nursing, this fact can cause mistakes in the assistance offered, as well as in harms to the patient and the institution.

When the specific components of the Pittsburgh Sleep Quality Index were observed, the domains of sleep quality and sleep disorders presented statistical significance with the type of hospital (p-value = 0.030 and <0.001, respectively) and the time working at the institution (p-value <0.001).

The first component is assessed by the professionals’ perception as to their sleep quality, with 51% reporting having a good or very good sleep. These data contradict Pittsburgh final classification and corroborate a study developed in Colombia, where 85.7% of the professionals reported having good and very good sleep quality, although they presented poor sleep quality in the final classification of the instrument^(24)^. It is emphasized that the better assessment can be related to the adaptation to the practice of the service, to working in shifts and to poor quality of sleep as the routine of the activities performed.

As for the type of hospital, in the Brazilian reality, the public institutions face difficult situations, such as insufficient material, and human resources and equipment, lack of beds and overcrowding in the services, which many times lead to professional illnesses^(35)^. In addition to that, despite the improvement in the quality and quantity of the health services, the pattern centered on primary and emergency care overloads the hospital services with high work demands^(36)^. These situations impose limitations to the competence and freedom of the professionals, since they are subjected to stress, suffering and poor working condition, with the objective of performing quality health care^(35)^, at the same time that they have stability as a factor that keeps them in the same employment contract.

In another study conducted in public hospitals in China, presence of poor sleep quality among nurses was obtained, with 68.8% of those that worked in high-complexity tertiary institutions presenting severe sleep disorders associated with great service demand, a greater number of patients to care of in the night shift and absence of recognition of the work performed^(21)^.

The presence of sleep disorder is assessed by the sum of 9 questions of the instrument that emphasize situations that can disturb sleep, being considered inadequate when exceeding 10 points. In this study, 27.6% of the professionals were characterized with some sleep disorder, being associated with the public hospital (p-value<0.001) and with longer working time (p-value<0.001). Regarding the hospital component, a study conducted in a regional Colombian institution obtained 10.2% of the professionals with such disorders, attributed to the environmental characteristics of their home and work^(24)^. Similarly, a research study carried in South Korea with nurses who worked in shifts obtained 50% of the professionals with sleep disorder, with 28% of them presenting severe sleep changes, fatigue and depression associated with the work in hospital units^(36)^.

A study conducted in Iran obtained a lower prevalence of sleep disorders, in which only 7.4% of the professionals presented severe insomnia, especially in night-shift workers^(23)^. A research study conducted in Sweden with newly graduated nurses obtained presence of sleep changes related to the concerns and persistent thoughts about work, in addition to a high load of activities^(28)^.

An integrative literature review study on the current knowledge and attitudes in relation to the impact of sleep disorders on health and on the cognitive functions among the Nursing team members in Europe showed that 30% to 70% of the nurses sleep less than six hours before the shift. Regarding the cognitive effects of sleep deprivation, it was observed that it impairs the performance of tasks that require prolonged and intensive attention, which increases the number of errors when serving the patient^(37)^.

It is also highlighted that the night shift, both with fixed and alternate working hours, is harmful to the professionals’ health, as it alters the productions of melatonin^(38)^, reduces work safety, productivity, performance and quality of life^(39)^, being related to gastrointestinal problems, pain in the back and neck, fatigue, depression, tiredness, stress, cardiovascular diseases and early mortality^(4,31)^. In addition, the 12hour working day followed by 36 hours of rest makes it easier for the employee to have a second job^(23)^. Having more than one employment contract generates extensive working days, with partial or nonexistent rest, which favors poor sleep quality.

In addition to that, with longer time working at the hospital institution, the development of skillful care is observed; however, with the professional experience, there is longer time of exposure to occupational stressors, which facilitates the development of sleep disorders^(22,24)^ and can wrongly contribute to reducing the perception of the need for protection against accidents and the understanding about one’s own health status^(35)^.

Sleep latency is assessed by the time needed to fall asleep, from the wakefulness stage to total sleep and, in this study, statistical significance was observed with having a job (p-value<0.001) and longer working time (p-value = 0.001). In addition to that, the mean time to fall asleep referred was 31.9 minutes, with a mean of 6.5 sleep hours. In a research study conducted in São Paulo (Brazil), it was found that 35.29% of the professionals reported needing more than 30 minutes to fall asleep^(23)^, as well as in study carried out in China, which obtained a higher latency among the professionals^(40)^ and a research study conducted in Colombia, in which 34% of the professionals needed more than 28 minutes to fall asleep^(24)^.

It is also known that sleep is influenced by hemodynamic conditions, temperature, environmental sounds, social activities and exposure to light. The latter has an important effect on the initiation and maintenance of this process, since artificial light alters the circadian and sleep-wake cycles^(41)^.

Furthermore, a study shows that there is large exposure to artificial light in the general population, with a constant increase in the last years, which, along with the environmental working factors, influences sleep quality^(31)^. It is suggested that the professionals with a job expose themselves more to artificial light during their leisure time, which, together with work exposure, contribute to difficulty in falling asleep.

In this study, statistical significance between the working shift and sleep duration was observed (p-value<0.001), in which the period of the day demonstrated higher mean points, suggesting that these professionals present fewer resting hours. However, national and international surveys obtained sleep disorders with worse quality in the night period^(1,2,10,26,28,33,35,42)^.

It is also pointed out that the anxiety and stress levels have an effect on the sleep assessment tool, as well as on the quality and duration of the physiological process. In this way, professionals of the day shift present more accentuated work routines, which contributes to more frequent awakenings at night, insomnia and less sleep time^(35)^.

The usual effectiveness of sleep is assessed in the question on how many hours were slept in relation to the time in bed, with white skin color (p-value = 0.010), two or more jobs (p-value = 0.040) and more than 40 working hours (p-value = 0.030) presenting higher mean scores in this study.

It is also pertinent to state that, despite the economic growth of the Nursing profession, the submission to long working hours and the duplicity of jobs are similar to the medical profession, which intensifies the risk of illnesses, dissatisfaction with sleep and insomnia^(43)^. A study carried out in China with physicians and nurses found that the workers presented very different sociodemographic and occupational characteristics and that the nurses had significantly greater mental health problems and sleep changes than the other workers^(42)^.

It is also contemplated that, in case of more than one employment contract and extensive workloads, the time devoted to sleep becomes scarce, the secretion of cortisol and the central temperature of the organism increase, with a reduction of melatonin and a consequent decrease of sleep efficiency and its duration^(44-45)^. A study developed in hospitals from Ontario, Canada, highlights that the extensive working days and the increasing number of overtime hours in Nursing exert an influence on the well-being of the patient, on the finance of the health institutions, on satisfaction and on the nurses’ health. This fact was justified by financial gain, career development, assistance offered to the colleagues and to maintain care continuity^(44)^.

Regarding the long working days, they were also observed in a study developed with nurses from Thailand, in which 79.34% of the participants reported an 8-hour workload, although they worked a mean of 58.82 hours during the week. In addition, 32.81% of the professionals indicated low job satisfaction and only 54.4% reported having adequate sleep^(45)^. As for the significant difference between skin color and the specific component, no studies similar to the result obtained were found in the literature.

Regarding the use of sleep medications, it was reported by 33.7% of the professionals, with varying frequency during the week. The specific component showed a significant difference with the public hospital (p-value = 0.030), females (p-value = 0.040), one job (p-value = 0.010) and longer time in the institution (p-value = <0.001), corroborating a research study carried out with the Nursing team from a public hospital, in which 51% of the participants used sleep medications^(46)^. As for a study carried out in Colombia, it was found that only 4.1% of the professionals used sleep medications^(24)^, similarly to another research study with nurses from Ethiopia, in which 24.6% of the participants made use of some medication to improve sleep quality^(27)^.

It is contemplated that the professionals justify taking a medication due to stress, excessive workload, poor working conditions and poor sleep quality. In addition, self-medication is considered a phenomenon that occurs more frequently among women, with high schooling and with facilitated access to the drugs^(27,46)^, a profile similar to the Brazilian.

As for the daytime dysfunction component, assessed by the question on remaining awake in routine tasks and excited to carry out the activities, a statistical significance with the hospital variable (p-value = 0.010) was observed, with a higher value for the public institution, suggesting greater sleepiness during the day in the professionals from the public service and poor subjective sleep quality.

Regarding the limitations of this research, the cross-sectional design stands out, which prevents data generalization and the monitoring of the changes developed by the professionals, as well as the noninvestigation of the individuals away from work. In addition to that, it was not the objective of the study to assess the rest conditions and social support in the work environment, variables that can influence the development of mental disorders and sleep changes. It is also noted that the sample was mostly composed of nursing technicians, as well that the instruments are self-applied and were made available to the participants for completion, with return scheduled at a later date. Such criteria can contribute to bias, perhaps affected by the interest and attitudes of the research participants.

The prevalence of sleep changes in the Nursing team corroborates national and international research studies, demonstrating that the lack of adequate sleep can contribute to professional illnesses and to worse Nursing care quality. Thus, it is believed that the data presented may collaborate to the understanding of the work dynamics of the Nursing professionals, lead to management changes, in order to monitor health changes and favor the elaboration of promotion and prevention programs for this population group.

However, the health policies, implemented by the managers, must propose actions that aim at to protect and to promote of the workers’ mental and physical health, reducing the adverse events in care, high rotation in the services, the increase in the hospital costs and dismissals associated with professional illnesses^(3,5,24)^.

## Conclusion

*A priori*, it is contemplated that poor sleep quality and sleep disorder were prevalent in the sample and, when the sociodemographic variables were observed, the presence of common mental disorder showed to be a factor associated with sleep changes. In addition to that, among the sociodemographic and occupational data, the public hospital, being female, white skin color, having a job, working more than forty hours a week, longer working time in the institution, and the night shift showed an association with the assessment of sleep quality.
